# Degradation of Aflatoxins by Means of Laccases from *Trametes versicolor*: An In Silico Insight

**DOI:** 10.3390/toxins9010017

**Published:** 2017-01-01

**Authors:** Luca Dellafiora, Gianni Galaverna, Massimo Reverberi, Chiara Dall’Asta

**Affiliations:** 1 Department of Food Science, University of Parma, 43124 Parma, Italy; luca.dellafiora@unipr.it (L.D.); chiara.dallasta@unipr.it (C.D.); 2 Department of Environmental Biology, Sapienza University, 00185 Rome, Italy; massimo.reverberi@uniroma1.it

**Keywords:** aflatoxins, biotransformation, enzymatic detoxification, laccase, mild technologies, food safety, mycotoxins mitigation

## Abstract

Mycotoxins are secondary metabolites of fungi that contaminate food and feed, and are involved in a series of foodborne illnesses and disorders in humans and animals. The mitigation of mycotoxin content via enzymatic degradation is a strategy to ensure safer food and feed, and to address the forthcoming issues in view of the global trade and sustainability. Nevertheless, the search for active enzymes is still challenging and time-consuming. The in silico analysis may strongly support the research by providing the evidence-based hierarchization of enzymes for a rational design of more effective experimental trials. The present work dealt with the degradation of aflatoxin B_1_ and M_1_ by laccase enzymes from *Trametes versicolor*. The enzymes–substrate interaction for various enzyme isoforms was investigated through 3D molecular modeling techniques. Structural differences among the isoforms have been pinpointed, which may cause different patterns of interaction between aflatoxin B_1_ and M_1_. The possible formation of different products of degradation can be argued accordingly. Moreover, the laccase gamma isoform was identified as the most suitable for protein engineering aimed at ameliorating the substrate specificity. Overall, 3D modeling proved to be an effective analytical tool to assess the enzyme–substrate interaction and provided a solid foothold for supporting the search of degrading enzyme at the early stage.

## 1. Introduction

Mycotoxins are low-molecular weight molecules produced as secondary metabolites by several species of fungi. They may enter the feed and food production chains worldwide upon the infection of crops and commodities intended for animal and human consumption. The contamination of food and feed by mycotoxins poses major concerns for the public health and welfare as the dietary exposure may cause disorders, dysfunctions and alterations of physiological states in both humans and animals [1,2].

Many countries have adopted regulations to reduce the possible dietary intake, thereby preserving the health of animals and consumers (for Europe: EC No 1881/2006, EU No 165/2010, EU No 105/2010). However, the allowed levels of contamination are not harmonized among countries, and this may cause trade frictions at the global level. De facto, the management of risks related to foodborne mycotoxins must consider several factors and have to reach controversial socio-economical tradeoffs, being primarily influenced by the availability of a secure food supply. The developing areas are the most damaged in terms of health and international exchanges as the contamination levels commonly found in traded commodities do not often comply with those enforced by industrialized countries, also considering that low-income countries lack regulatory actions and monitoring plans. As an example, the restrictions of the European Regulation cause huge economic losses to Africa, unreasonably exceeding the limits to effectively safeguard the public health in the European countries, as commented by the past Secretary-General of the United Nations, Kofi Annan [3].

On this basis, the mitigation of mycotoxin content in food and feed is a critical foothold to address the forthcoming challenges in view of the sustainability and global trade. On the one side, the reduction of contamination levels in food and feed can effectively ameliorate the health and welfare of both humans and animals. On the other side, the implementation of cost-effective strategies for recovering contaminated food and raw materials after spoilage may concretely allow the weaker markets to reenter the global trade. In this scenario, the development of affordable and straightforward strategies for mitigating mycotoxin content is definitely a major task for the scientific research.

A wide number of strategies for the control and mitigation of mycotoxins content in food and feed are currently under consideration. For instance, the strategies of biocontrol with non-toxigenic fungi, conventional breeding and genetic engineering are promising methodologies aimed at preventing the accumulation of mycotoxins on the field at the pre-harvest level [4]. Instead, food processing, physical methods (e.g., irradiation and adsorption) and microbial/biochemical transformation of mycotoxins to non- or less toxic compounds may act post-harvest on raw material or intermediate products [4]. The main advantage of the latter approach on the former is the possible application to low- or non-compliant food batches to reduce and/or reuse wastes. Among these, the enzymatic transformation seems to be the most promising tool for the mitigation in situ [5].

The high-throughput search and optimization of effective enzymes to be used for mitigation is thus of primary interest, even though highly challenging and time-consuming. Typically, the first steps in the conventional large-scale research process of active enzymes inevitably include the coarse-grained selection of candidate enzymes, which drastically depends on the realistic affordability of proteins in sufficient amounts and/or in the active forms. Commonly, this hardly complies with the rational criteria of exclusion in terms of possible effectiveness. However, the search of putative active enzymes in the early steps can be effectively boosted in a straightforward and cost-effective manner by using screening procedures in silico. Indeed, the upstream use of computational approaches to screen the libraries of candidate enzymes may support a wide-ranging and evidence-based selection of those enzymes to be investigated experimentally. In particular, the use of the 3D modeling by means of the computational estimate of the interaction at the enzymes binding site deepens the structural aspects underlying the enzyme–substrate interaction and succeeds in identifying substrates (e.g., ref. [6,7]). Hence, it can be a reliable tool for providing the rational and evidence-based hierarchization of enzymes on the basis of the computed capability to allow a favorable arrangement of substrates.

In this framework, the present study addressed the enzymatic degradation of aflatoxins (AFs) by laccase enzymes from *Trametes versicolor*. AFs are difuranocoumarin derivatives produced by *Asperigillus sect. Flavi* that can be found as contaminants primarily in cereals, maize, oilseeds and nuts [8]. AFs are mutagenic, genotoxic and carcinogenic compounds that cause both acute and chronic health effects [9]. Among the 20 AFs identified so far, aflatoxin B_1_ (AFB_1_) is the most widespread and harmful in terms of both acute and chronic toxicity [10], while aflatoxin M_1_ (AFM_1_)—its major hepatic metabolite in mammals—raises concern as it can be found in milk and dairy products [11]. In recent years, severe outbreaks related to aflatoxin contamination in feed have been reported in Europe, mainly in the Mediterranean and Balkan areas [12]. Besides health concerns, these events caused significant losses in terms of veterinary costs and managing of incompliant feed and milk batches.

Laccases are multi-copper containing enzymes capable of performing one electron oxidation of a broad range of substrates [13]. The laccase enzymes from *T. versicolor* have been identified as a promising route for the low-cost and effective reduction of AFB_1_ content [14,15], while the use of these enzymes has been never considered before to degrade AFM_1_. Moreover, the strategies commonly rely on the use of mixtures of the various laccase isoforms. The relative activity of the various isoforms and the structural aspects of aflatoxin interaction have not been elucidated yet, making the design of rational strategies based on selected isoforms difficult.

**Figure 1:**
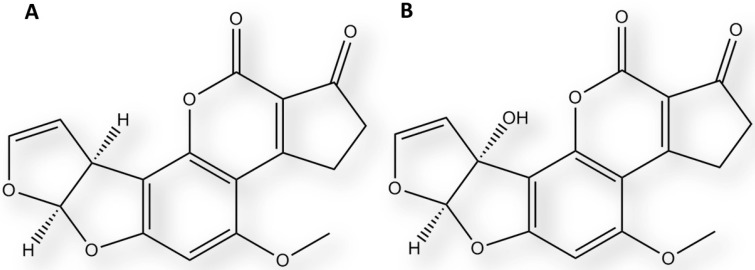
Chemical structure of aflatoxin B_1_ (**A**) and aflatoxin M_1_ (**B**).

As a proof of concept, the present work aimed at modeling the interaction of AFB_1_ and AFM_1_ (Figure 1) with three out four laccase isoforms from *T. versicolor* (namely, the beta, delta and gamma isoforms) in order to find out possible differences among the enzymes in terms of pocket–ligand recognition. The complementarity of both AFB_1_ and AFM_1_ towards the various catalytic sites has been assessed using a previously validated structure-based molecular modeling workflow based on docking simulation and rescoring procedures. The pharmacophoric analysis of catalytic sites and the comparison of structures and sequences of the various isoforms have been done to better understand the basis of the enzymes–substrates interaction at a molecular level.

## 2. Results

### 2.1. Sequence Analysis and Pocket Anatomy

**Figure 2:**
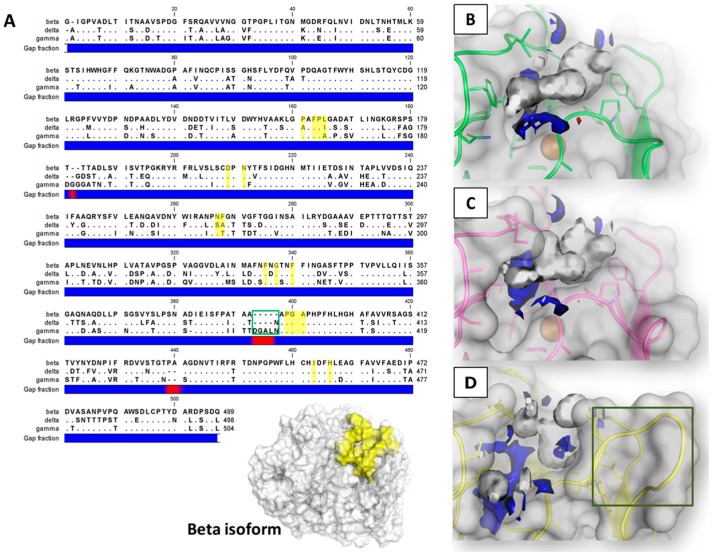
The sequence alignment (**A**) and pharmacophoric analysis of beta (**B**), gamma (**C**) and delta (**D**) laccase isoforms from *T. versicolor*. In the sequence alignment (box **A**), dots represent matching residues while dashes indicate gaps (**red** spots in the gap fraction **blue** bar). Residues of the binding site are highlighted in **yellow** while the **green** box indicates an extended region of the gamma isoform lining the catalytic site. The overall 3D structure of the beta isoform is also reported to provide localization of the binding site (colored in **yellow**). In the pharmacophoric analysis (boxes **B**, **C** and **D**), **white**, **red**, and **blue** contours identify regions sterically and energetically favorable for hydrophobic, H-bond acceptor, and H-bond donor groups, respectively. Spheres indicate Cu ions. The **green** box indicates the extended region of the gamma isoform.

Laccases from *T. versicolor* show a globular structure with approximate dimensions of 70 × 50 × 50 Å with a topology consisting mainly of antiparallel β-barrels [16]. The overall amino acids sequence alignment of the laccase enzymes (from here on referred to as models) revealed that the delta and gamma isoforms showed, respectively, 71.6% and 71.8% of the identity with the beta isoform, and 77% between themselves (Figure 2A).

Concerning the primary structure, the sequence alignment revealed that the binding site of the beta and delta isoforms appeared fairly comparable to each other (12 residues out of 16 are conserved). Instead, the gamma model showed a higher divergence mainly due to the presence of an extend loop with five and four additional residues in respect to the beta and delta isoforms, respectively (Figure 2A,D).

The pharmacophoric analysis of the various binding site environments revealed that all the pockets appeared prevalently hydrophobic, as the hydrophobic environment turned out to exceed in extension the hydrophilic one, with a limited capability to receive polar groups, wherein H-bond donors groups were found to be more energetically favored than the H-bond acceptor ones (Figure 2B–D). In addition, concerning the distribution of the polarity of the space available for ligands, the binding sites of beta and delta isoforms were found to be more similar to each other, while the site of the gamma isoform was found to be less hydrophobic with a more extended volume energetically able to receive H-bond donor groups. 

### 2.2. Assessment of Procedure Reliability

The in silico investigation of AF-laccase interactions relied on the assessment of pocket–substrate complementarity through the coupling of docking simulation to calculate the binding architecture with re-scoring procedures using the HINT (Hydropathic INTeractions) scoring function for the careful estimation of the energetic contributions of the binding event (see Section 5 for further details). Such a procedure already proved to be an effective strategy for investigating the protein–ligand complex formation and the biological activity of chemicals, [17,18], and succeeded in identifying enzymes substrates as well [6]. However, the case-by-case assessment of procedural performances is required to prove the fit-for-purpose reliability among the diverse case studies [19].

Therefore, in the present work, the two benchmark laccase substrates ABTS (2,2′-azino-di-(3-ethylbenzothiazoline)-6-sulfonic acid) and 2,6-dimethoxyphenol [20] have been chosen to assess the case-specific reliability of the computational procedure. Specifically, the procedure has been validated assessing the capability to properly rank the reference compounds in accordance with the experimental data from the literature, and for the capability to reproduce the 3D binding architecture observed in the crystallographic structures available so far. The K_m_ values indicate the enzymes affinity for substrates. Thus, the K_m_s have been chosen among the various biochemical parameters for the comparison with the computed scores since the HINT scoring may correlate the pocket–ligand affinity proportionally [6,21,22,23,24,25].

ABTS showed a clearly defined rank of K_m_s among the various isoforms, with the following order: 88 μM in beta < 359 μM in gamma < 2262 μM in delta [26]. As shown in Table 1, the computational ranking of ABTS among the various laccase isoforms was consistent with that observed experimentally.

Moreover, while the beta isoform *wild type* shows a higher affinity for ABTS than for 2,6-dimethoxyphenol, previous studies demonstrated that the D206A mutation at the level of binding site causes the inversion of ranking [20,27]. As reported in Table 2, the computational ranking of ABTS and 2,6-dimethoxyphenol within both the mutated and *wild type* enzyme was consistent with that observed experimentally.

**Figure 3:**
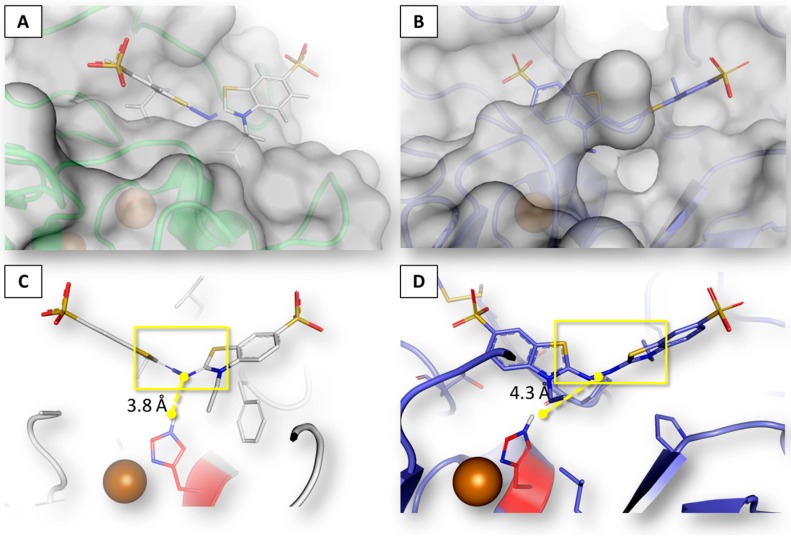
Binding architecture of ABTS. Proteins are represented in cartoon and surface, ABTS and residues of binding sites are represented in sticks. Cu at the T1 site is represented by the **red** sphere and the catalytic histidine is colored in **red**. ABTS electron donor region is highlighted with the **yellow** box, while interatomic distances are indicated by **yellow** dashed lines. (**A**) calculated surface interaction with beta laccase from *T. versicolor*; (**B**) crystallographic surface interaction with CotA laccase from *Bacillus subtilis* [29]; (**C**) detail of binding architecture with beta laccase from *T. versicolor*; and (**D**) detail of binding architecture with CotA laccase from *B. subtilis* [29].

In order to assess the reliability of the procedure in predicting the binding architectures, the computed pose of ABTS within the beta isoform was compared with the crystallographic one within the orthologous laccase enzyme from *Bacillus subtilis*, as no structures of laccases from *T. versicolor* are available so far. They can be consistently compared as orthology allows taking the functional conservation in different species for granted (i.e., the catalytic reaction and the overall organization of substrates in this case) [28]. It should be kept in mind that laccase enzymes catalyze the one-electron oxidation of substrates involving a Cu ion (at the so-called Cu T1 site), coupled to the four-electron reduction of molecular oxygen to water at the tri-nuclear Cu cluster [27]. The catalytic histidine lining the binding site, which may interact with substrates, mediates the electron transfer. For a proper transfer, the distance of the electron–donor regions of substrates from the receiving histidine cannot exceed the 5 Å [26]. The inspection of the crystallographic pose of ABTS revealed that the electron–donor region occupies the deepest region of the binding site, closely arranged to the catalytic histidine, while sulfonic acid groups protrude outside. Therefore, the geometric reliability of the in silico procedure was established by assessing the capability to properly reproduce such binding architecture. As showed in Figure 3, the overall computed organization of ABTS turned out to be consistent with the crystallographic pose and the arrangement of the electron–donor region has been correctly predicted as well, being posed within the 5 Å needed for undergoing the reaction [27].

### 2.3. Interaction of Aflatoxin B_1_ and M_1_ within Beta, Delta and Gamma Laccase Isoforms

The scores of interactions between AFs and laccase isoforms are reported in Table 3. AFM_1_ was found able to interact favorably with all the laccase isoforms herein considered. AFB_1_ favorably interacted instead with beta and delta, but not with gamma, as the pharmacophoric requirements were not satisfied (see below).

**Figure 4:**
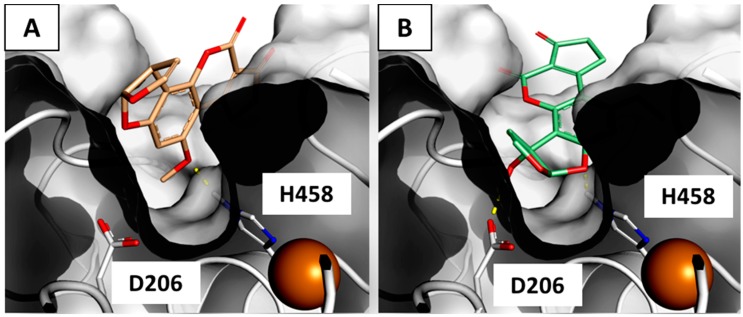
Binding architecture of AFB_1_ (**A**) and AFM_1_ (**B**) within the beta isoform. Proteins are represented in with cartoons and cut surfaces, and ligands and amino acids side-chains are represented with sticks. The Cu ions are represented with spheres. **Yellow** dotted lines indicate H-bonds.

The close inspection of the computed poses revealed that AFB_1_ adopted comparable binding architectures within both the beta and delta laccase isoforms, wherein the methoxy moiety was oriented toward the bottom of the binding sites. Both the interactions were mainly driven by hydrophobic/hydrophobic interaction, in accordance with the marked hydrophobic environment of the pockets. Indeed, a unique polar contact was found, wherein the oxygen of the methoxy group engaged the His458 (according to the amino acids’ numeration of the beta isoform) with a hydrogen bond (Figure 4A). AFM_1_ engaged the His458 with the oxygen on the difuran ring and used the additional hydroxyl group for engaging Asp206 (according to the amino acids’ numeration of the beta isoform), thereby embedding much more into the catalytic sites (Figure 4B) and retracing the mode of interaction proposed for phenolic substrates [27]. In addition, the formation of the additional hydrogen bond was responsible of the higher score recorded for AFM_1_ within all of the laccase isoforms herein considered. Conversely, AFB_1_ was unable to sink into the site to the extent of AFB_1_ since the hydroxyl-free difuran moiety did not find the energetic favors for being close to the aspartate side chain.

**Figure 5:**
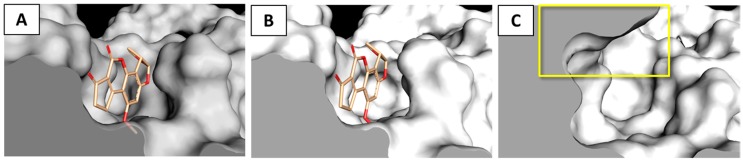
Details of ligand binding site of laccase isoforms. Proteins are represented with cut surfaces and ligands with sticks. The **yellow** box indicates the additional volume in the gamma isoform binding site due to the presence of the extended loop. (**A**) AFB_1_ within the beta isoform pocket; (**B**) AFB_1_ within the delta isoform pocket; and (**C**) gamma isoform pocket.

The incapability of AFB_1_ to positively interact with the gamma isoform may be explained by the diverse capability of AFB_1_ and AFM_1_ to sink within the catalytic site, where AFM_1_ was found closer to the bottom of the pocket. Indeed, the additional loop at the entrance of the binding site of the gamma isoform redefined markedly the available space for ligands in the upper portion of the pocket (Figure 5) and prevented the proper accommodation of AFB_1_.

## 3. Discussion

The mitigation of mycotoxin content in food and feed is undoubtedly a major task for safeguarding health and global trade. Besides the use of good agricultural practices and crop breeding, the biological control of toxin accumulation in the final products by acting at pre- and post-harvest levels and during food processing proved to be an effective strategy [4]. In this framework, the search for enzymes able to convert mycotoxins into non- or low-toxic products has been the object of a growing number of studies. The main advantage of using enzyme-based strategies is the possibility to act at the different stages of food and feed production chains. Indeed, enzymes are currently considered as food/feed additives or agents during the from-field-to-fork pathway, and they are aimed at reducing the carryover and accumulation in the final products [30]. Nonetheless, while the safety and security of genetically modified organisms for health and environment are under a heated scientific debate, the genome engineering by introducing effective enzymes in susceptible hosts or detoxifying microorganisms can be thought as a possible (future) strategy (e.g., ref. [31,32]).

In this scenario, novel strategies for a more effective search of mycotoxin-degrading enzymes should be implemented. The upstream investigation via in silico approaches can be a straightforward choice to significantly extend the explorable space in the early stage providing reliable and informative insights on the enzymes–substrate interaction, also in the view of the evidence-based preliminary hierachization of candidates for the experimental trials.

As a proof of concept, in the present work, we addressed the case study of the interaction between AFs and laccase enzymes from *T. versicolor*. The tertiary structures of gamma and delta isoforms, which are not structurally resolved up to now, were obtained through the homology modeling on the crystallographic structure of the homologous beta isoform (further details are reported in Section 5). On the basis of the fit-for-purpose validation, the 3D receptor modeling turned out to be an effective strategy for estimating the enzyme–substrate interaction for all of the isoforms under investigation. Actually, the reliability of 3D models that were derived from the primary structure of proteins is among the most relevant outcomes pinpointed herein. Indeed, the possibility to use the primary structure for deriving reliable 3D libraries of enzymes may significantly expand the explorable space of research beyond the enzymes that are commercially available or structurally and functionally known so far. In this respect, the advances in structural biology and the whole-genome sequencing data are providing a growing number of high-resolution structures to derive 3D models and a wealth of newly identified sequences of putative proteins for an even more wide-ranging screening.

The degradation by means of laccase enzymes from *T. versicolor* is considered among the most promising and cost-effective enzymatic strategies for the mitigation of AF content [14]. Typically, the development of enzyme-based strategies requires the precise understanding of the specific enzymes–substrate activity, especially in the presence of different isoforms, in order to isolate the most suitable for the purpose. To this end, it is mandatory to gain knowledge on the substrate–enzyme interaction from a structural perspective, thereby understanding in-depth the mechanism of catalysis and deciphering the reasons underlying the formation of degraded products. Taken as a whole, this background of knowledge may reduce the extent of the try-and-error timeframe during the optimization of degradation processes. However, the relative effectiveness of the various laccase isoforms from *T. versicolor* is still unknown, as only unspecified mixtures of the various isoforms have been assessed up to now. Furthermore, the structural organization of AFs within the laccase binding sites has not been elucidated yet. Overall, this scenario eventually makes it hard to refine more effective strategies based on selected enzymes. In this context, the present work investigated the arrangement of AFB_1_ and AFM_1_ within the binding site of the beta, delta and gamma laccase isoforms with the aim to find out some possible differences in the accommodation of AFs in the catalytic sites. On the basis of our findings, relevant structural differences between gamma and the other isoforms have been pointed out in terms of accessibility of the catalytic site. In fact, AFB_1_ were found able to accommodate within beta and delta, but not within gamma due to the extension of a loop lining the binding site. In particular, the re-shaping of the upper portion of the pocket was responsible for the inappropriate accommodation of AFB_1_ since the hydrophobic difuran moiety has been unfavorably arranged too close to the hydrophilic space at the bottom of the pocket. Notably, the pathway of entrance toward the catalytic site of enzymes may strongly influence the reaction yield and commonly concur to determine the substrate specificity [33,34]. Specifically, it has been previously reported that mutation at the C-terminus of orthologous fungal laccases may affect the enzyme activity influencing the accessibility to the binding sites [35,36]. Accordingly, this feature can be accounted for gaining more specificity for the laccase enzymes from *T. versicolor* since the low substrate specificity is the major drawback in the application on real food and feed matrices. Indeed, laccases can degrade a wide spectrum of compounds, including a wealth of healthy low molecular weight food and feed constituents (e.g., polyphenols) [37], thus possibly causing an overall pauperization of the treated products. Therefore, the engineering of such a loop might be a straightforward strategy to modulate the substrate specificity of laccases without significantly altering the binding site and preserving as much as possible the catalytic environment. To this end, the gamma isoform can be considered as the most suitable among the various isoforms holding an extended loop at the entrance of the catalytic site, which might be accounted for mutations, while the other isoforms have a direct access.

AFM_1_ was found able to positively interact within the catalytic sites of all the isoforms herein considered. In this case, the presence of the hydroxyl group on the difuran moiety facilitated a deeper sinking into the pocket that allowed the accommodation also within the gamma isoform. Notably, AFM_1_ recorded higher scores than AFB_1_ within all of the catalytic sites due to the formation of an additional hydrogen bond. The gain of enthalpy may be responsible for an increase in substrate–enzyme affinity and, eventually, may cause a higher degrading yield. In this respect, the full degradation of AFM_1_, by laccases from the edible mushroom *Pleurotus pulmonarius*, was reported by Loi and coworkers [38], thus supporting the strong degradation of hydroxylated forms in accordance with the well-known activity of laccases on poly-phenolic molecules [27].

In the framework of effectively reducing the content of toxicants, the formation of more toxic by-products must be carefully avoided. In this respect, a decreased (geno)toxicity for AFB_1_ after treatment with laccases has been already reported. The modification at the level of difuran moiety—which is responsible for the toxic action—has been proposed as the possible mechanism [15]. However, neither the exact chemistry of the degrading event nor the structure(s) of reaction product(s) have been elucidated so far. The computed architectures of binding revealed that both AFs structures (including the difuran moieties) were found almost entirely arranged within the proper range of distance from the catalytic hystidine to undergo oxidation. Thus, some other parts of the AFB_1_ molecules might undergo modifications. Moreover, it is worth mentioning that AFM_1_ showed some differences in the pattern of interaction with respect to AFB_1_ as the additional hydroxyl group on the difuran moiety interacted directly with the catalytic core. Accordingly, AFM_1_ might undergo the same degrading route of phenolic compounds via the electron/proton transfer mechanism [27], which is instead less likely to occur for AFB_1_ due to the lack of the additional hydroxyl groups. Therefore, differences in terms of sites, type of modification and chemical structures of products between AFB_1_ and AFM_1_ cannot be excluded throughout and should be carefully evaluated to rule out the formation of possible toxic byproducts.

## 4. Conclusions

In conclusion, the in silico simulations proved to be effective analytical tools to investigate the enzyme–substrate interaction, correlating with the affinity of binding. Specifically, the 3D modeling approach provided, for the first time, structural insights on the laccase–aflatoxin interaction, which may be useful for the evidence-based hierarchization of enzymes to be used in further experimental trials.

The modeling of laccase enzyme has been shown to be reliable from previous works (e.g., ref. [39,40]). However, the in silico screening of multiple laccase isoforms in the framework of AF control has been never used before. In more detail, AFM_1_ was found to be able to arrange positively within all the isoforms herein considered, while AFB_1_ was able to arrange within beta and delta but not within gamma. Accordingly, the low degradation yield of AFB_1_ by laccase gamma can be hypothesized. Furthermore, AFB_1_ and AFM_1_ showed different binding architectures in arranging within laccase catalytic sites. Therefore, it cannot be excluded that AFB_1_ and AFM_1_ undergo different modifications in different regions of the molecule, thus forming degraded products that are chemically different. This might also cause the differential formation of toxic by-products. On the other hand, the effects of the extended loop of the gamma isoform in diversifying the enzyme–substrate recognition can be accounted for developing future strategies to modulate the substrate specificity. Taken together, these results may provide a basic foothold for addressing future studies from a more informed perspective.

Finally, it is worth mentioning the degradation of AFM_1_, which is the main mammal metabolite. The carryover phenomenon of this metabolite in dairy products poses serious health concerns, and, nowadays, the strategies for reducing the contamination levels in milk and derived products primarily act on reducing the consumption of AFB_1_-contaminated feed by dairy animals. However, the use of AFM_1_-degrading enzymes on milk might be an additional strategy to further mitigate the contamination level of AFM_1_ in milk itself and dairy products.

## 5. Materials and Methods

### 5.1. Homology Modeling and Sequence Analysis

The crystallographic structure of the enzyme beta laccase from *Trametes versicolor* (PDB code 1KYA [16]) was the template for the homology modeling of gamma and delta isoforms. The Modeller software, version 9.14 (copyright © 1989-2016 Andrej Sali; maintained by Ben Webb at the Departments of Biopharmaceutical Sciences and Pharmaceutical Chemistry, California Institute for Quantitative Biomedical Research, Mission Bay Byers Hall, University of California San Francisco, San Francisco, CA, USA) was used [41]. The D206A beta isoform model was obtained by manually editing the wild type structure with the software Sybyl, version 8.1 (Certara USA, Princeton, NJ, USA). For sequence analysis, a local pairwise alignment was conducted by using the on-line tool EMBOSS-Water Pairwise Sequence Alignment (EMBL-EBI, Wellcome Genome Campus, Hinxton, Cambridgeshire, UK; http://www.ebi.ac.uk) and the Smith–Waterman algorithm was chosen.

### 5.2. Molecular Modeling

All protein structures and ligands were processed by using the software Sybyl, version 8.1 (Certara USA, Princeton, NJ, USA). All atoms were checked for atom- and bond-type assignments. Amino- and carboxyl-terminal groups were set as protonated and deprotonated, respectively. Hydrogen atoms were computationally added to the protein and energy-minimized using the Powell algorithm with a coverage gradient of ≤0.5 kcal (mol·Å)^−1^ and a maximum of 1500 cycles.

### 5.3. Pharmacophore Models

The ligand binding site was defined by using the Flapsite tool of the FLAP (Fingerprint for Ligand And Protein) software version 2.0 (Molecular Discovery Ltd., Borehamwood, Hertfordshire, UK; http://www.moldiscovery.com) [42], while the GRID algorithm [43,44] was used to investigate the corresponding pharmacophoric space. The hydrophobic (DRY) probe was used to describe the potential hydrophobic interactions, while the sp2 carbonyl oxygen (O) and the neutral flat amino (N1) probes were used to describe the hydrogen bond acceptor and donor capacity of the target, respectively. All images were obtained using the software PyMol version 1.7 (Schrödinger, New York, NY, USA; http://www.pymol.org).

### 5.4. Docking Simulations and Re-Scoring Procedures

The coupling of GOLD (Genetic Optimization for Ligand Docking), as docking software, and HINT [45], as rescoring function, was chosen on the basis of previous studies demonstrating the higher reliability of HINT with respect to other scoring functions in estimating the ligand binding free energies and evaluating the protein–ligand complex formation [19,23,46]. Software setting and rescoring procedures reported by Ehrlich were used [47]. In more detail, HINT score provides the evaluation of thermodynamic benefits of protein–ligand interaction, and relates with the ΔG° of complex formation [22,24,25]. Specifically, the empirical HINT scoring function implicitly considers enthalpic and entropic aspects of protein–ligand interaction using experimental Log Po/w measurements (partition coefficient for 1-octanol/water) as the basis of its force field. Indeed, from a mechanical point of view, the forces that drive the repartition of molecules between the two solvent phases also underline protein–ligand interaction, as well protein–protein interaction or ligand–ligand interaction. The HINT score is the sum of the all inter-atomic contributions from binding, thereby providing an empirical and quantitative estimate of the favors of the host–guest interaction from an atomic point of view. Thus, the higher the score, the more favored is the arrangement of ligands within the binding site [22,24,25,48]. The HINT equation is the following:

$$\mathrm{HINT}\;\mathrm{score}\;=\;\sum_{i}\sum_{j}b_{ij}=\sum_{i}\sum_{j}\left(a_{i}S_{i}a_{j}S_{j}T_{ij}R_{ij}+r_{ij}\right),$$

where *b_ij_* is the interaction score between atoms *i* and *j*, *a* is the hydrophobic atomic constant, *S* represents the solvent accessible surface area, *T_ij_* is a logic function assuming +1 or −1 values, depending on the nature of the interatomic interaction, and *R_ij_* and *r_ij_* are functions of the distance between atoms *i* and *j*. Further details on the basic theory of HINT can be found in ref. [49,50,51].

## Figures and Tables

**Table 1:** Comparison of experimental affinity and HINT (Hydrophatic INTeractions) scores of ABTS (2,2′-azino-di-(3-ethylbenzothiazoline)-6-sulfonic acid) within the various laccase isoforms.

Laccase Isoform	Experimental Affinity Rank ^1^	HINT Score
Beta	1	430
Gamma	2	206
Delta	3	104

^1^ As reported by Christensen and co-workers [26].

**Table 2:** Comparison of experimental affinity and HINT scores of ABTS and 2,6-dimethoxyphenol within the *wild type* and mutated form of beta isoform.

Laccase Isoform	ABTS		2,6-dimethoxyphenol	
	Experimental Affinity Rank ^1^	HINT Score	Experimental Affinity Rank ^1^	HINT Score
Beta *wild type*	2	430	1	500
Beta D206A	1	495	2	203

**^1^** As reported by Madzak and co-workers [20].

**Table 3:** HINT scores of aflatoxin B_1_ (AFB_1_)and aflatoxin M_1_ (AFM_1_)within the various laccase isoforms.

Laccase Isoform	HINT Scores	
	AFB_1_	AFM_1_
Beta	248	373
Gamma	−199	372
Delta	291	339
